# The Treatment of Fractures from a Common-Sense Point of View

**Published:** 1907-09-28

**Authors:** 


### THE TREATMENT OF FRACTURES FROM A COMMON-SENSE POINT OF VIEW.

### VI. FRACTURE OF THE CLAVICLE.

Fracture of the clavicle is a frequent but fortunately not a very severe injury. It is frequent, because the position of the bone is superficial, which exposes it to fracture by direct injury, and because the whole weight of the falling body is transmitted to it through the outstretched arm, so that fracture by indirect violence is common. It is rarely a severe injury because it is not often associated with the complications which accompany fractures of other long bones. Compound fracture of the clavicle, for instance, is most uncommon, because the skin over it is loose and the fascia covering it is strong; nor are the vessels which pass under it often injured, because they are protected by the subclavius muscle from injury by jagged ends of bone. It has, however, been known to have fatal results, and at least two historical characters, William III. and Sir Robert Peel, succumbed to the results of this injury.

The commonest form, and the only one in which there is any noticeable displacement, is a fracture at the junction of the two curves. If it be remembered that the normal action of the clavicles is to keep the shoulders up and back, that is to say, to prevent them from falling towards the middle line, the displacement which occurs when the clavicle is fractured will be easily understood. The moment the bone is broken the shoulder on that side tends to fall in, so that the outer fragment of the bone is carried downwards and forwards with its inner extremity tilted up. The inner fragment does not move from its normal position, being held in place by the rhomboid ligament, which is an exceedingly strong structure. In rarer cases the bone may be fractured at the sternal or at the acromial end, or between the conoid and trapezoid ligaments. These injuries are generally produced by direct violence, such as a fall on the point of the shoulder, and there is no displacement—an important point, because these conditions are easily overlooked.

#### Treatment.

Many methods of treatment have been suggested for fractures of the clavicle. Union appears to occur equally readily whatever form of treatment is adopted, and the usefulness of the arm is hardly ever impaired. In many cases the only difference between a good and a bad result is that the line of the bone after union is uniform and smooth in the one, while in the other there is an unsightly projection due to callus. It is especially important to avoid this in ladies who have to wear low-necked evening dress; and there is no doubt that in these patients the best line of treatment is the recumbent one. The patient is kept flat in bed, with a narrow pad under her back and a small pad in the axilla, the arm being bound to the side and the elbow brought forward. This position is maintained until the union is fairly firm, usually about two weeks; but it is a very tedious business, and the patients complain bitterly of it.

#### Sayre's Bandage.

In ordinary cases treatment by Sayre's method is usually adopted. In this a pad is placed in the axilla and a long piece of strapping is fixed to the arm, the end being stitched round it with the adhesive side externally. The loose end of the strapping is then brought round behind the body so that the arm is dragged backwards and is fixed to the chest. The elbow is now pulled forwards and fixed with a second piece of strapping which starts at the sound shoulder, is brought obliquely down across the back to the elbow of the injured side, and is brought forward across the chest to the sound shoulder again, thus fixing the forearm to the chest. The method answers quite well. When the elbow is brought forward after the first piece of strapping is fixed, the point of the shoulder is forced upwards and backwards, and the arm is thus kept in the position in which it rests when the clavicle is intact. But it has disadvantages. The strapping works loose when the patient gets about, and has to be reapplied one or more times before union is firm. And, again, it is very hard for the patient to wash himself; so that after a short time his condition becomes unpleasant, especially in the axillary region. As a matter of practical fact, one finds that however a fractured clavicle is treated the results are much the same, as long as the shoulder is kept up and back. A very simple and efficient way of doing this, which has none of the disadvantages of Sayre's method, is to use what are known as clavicle rings. These consist of rings of padded lint of such size that they encircle the arm at the shoulder joint. One is put on each arm, and the two are joined together behind by tapes, which are tightened until the shoulder is in the requisite position. Admirable results can be obtained by the use of this quite simple apparatus.

